# Factors affecting serum phenobarbital concentration changes in pediatric patients receiving elixir and powder formulations

**DOI:** 10.1186/s40780-021-00190-2

**Published:** 2021-02-02

**Authors:** Chihiro Shiraishi, Hiroko Matsuda, Toru Ogura, Takuya Iwamoto

**Affiliations:** 1 Department of Pharmacy, Mie University Hospital, 2-174 Edobashi, Tsu, Mie 514-8507 Japan; 2 Clinical Research Support Center, Mie University Hospital, Tsu, Japan

**Keywords:** Phenobarbital, Pediatric care, Elixir, Powder, Serum concentration

## Abstract

**Background:**

Phenobarbital (PB) is commonly used as elixir and powder formulations in pediatric care. Its dose adjustment is performed based on individual drug concentration monitoring. Few studies have comprehensively analyzed the variation factors for serum PB concentration. In this study, we retrospectively investigated the factors that influence serum PB concentration and assessed the impacts of dosage formulation and administration route.

**Methods:**

This retrospective cohort study covered clinical data from January 2007 to September 2019 at Mie University Hospital. The present study included 60 pediatric patients administered the elixir and powder of PB through oral route and enteral tube. Simple and multiple linear regression analyses were performed to identify the risk factors that affect the weight-corrected PB serum concentration/dose (C/D) ratio in pediatric patients. Six subgroups were also established according to the concomitant use of drugs that potentially inhibit PB metabolism, dosage formulation, and administration route to investigate the difference in the PB C/D ratio among the subgroups.

**Results:**

A significant regression equation to predict the PB C/D ratio was found through simple and multiple linear regression analyses, with an adjusted coefficient of determination of 0.53 (*p* < 0.001). Further, the concomitant uses of valproic acid (VPA) or amiodarone, which were the only two drugs seen in this study as potential inhibitors of PB, was found to have the greatest effect on the PB C/D ratio (standardized partial regression coefficient (β) = 0.543, *p* < 0.001). Furthermore, a significant difference in the PB C/D ratio was found between the subgroups classified by the concomitant use of VPA or amiodarone (*p* = 0.002). However, there were no significant correlations between the PB C/D ratio, dosage formulation, and administration route.

**Conclusions:**

The most influential factor on the PB C/D ratio was the concomitant use of VPA or amiodarone with PB. This result could provide an important perspective in pediatric drug therapy where elixir and powder formulations are administered via the oral route and enteral tube.

## Background

In 1912, phenobarbital (PB) was developed and released by Hauptmann [1] for the treatment of epileptic seizures [2, 3]. Presently, it remains one of the first-line drugs for the treatment of suspected neonatal seizures [4]. PB requires drug concentration monitoring to assess compliance, evaluate therapy response and adjust dosage. In pediatric medical care, dosage formulation is often switched between elixir and powder for several reasons, such as nonadherence and easy handling of dose adjustment with growth in pediatric patients. Previous investigations have demonstrated that serum PB concentration increased after the dosage formulation was switched from powder to elixir without a change in dose. In addition, the weight-corrected serum concentration/dose (C/D) ratio of PB in pediatric patients receiving PB elixir was reported to be significantly higher than that in pediatric patients receiving PB powder [5]. However, few studies have comprehensively analyzed the variation factors for serum PB concentration, including the dosage formulation and administration route.

In this study, we comprehensively analyzed the factors that affect the changes in serum PB concentrations, including the dosage formulation and administration route, in pediatric patients.

## Methods

### Patients

**Fig. 1:**
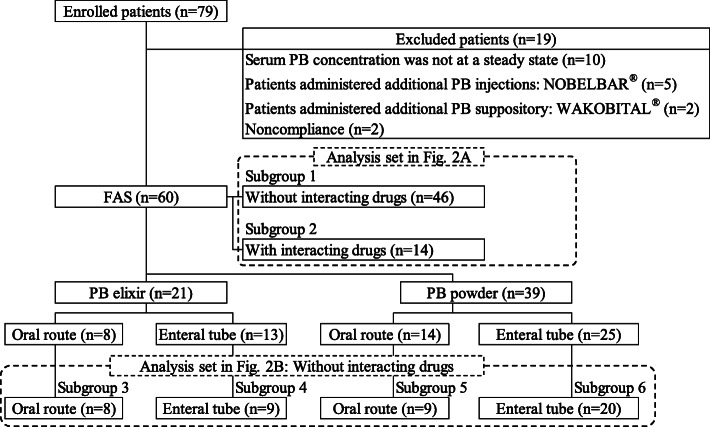
Flow chart of patient selection

This retrospective study included data from 79 pediatric patients from January 2007 to September 2019 at Mie University Hospital. Patients meeting the following inclusion criteria were enrolled: (i) received the elixir and powder of PB (PHENOBAL® POWDER 10% or ELIXIRS 0.4%, DAIICHI SANKYO Co., Ltd., Tokyo) orally and via an enteral tube, (ii) 0–15 years of age, and (iii) had one or more serum PB samples. Patient demographic data were obtained via a review of electronic medical records. Patients were excluded if their serum PB concentration was not at a steady state (*n* = 10), which was attained by repeated constant PB dose for more than 10 days based on the elimination half time of 2.5 days in the pediatric population [6]. Patients administered additional PB injections (NOBELBAR®, NOBELPHARMA Co., Ltd., Tokyo) (*n* = 5) or PB suppository (WAKOBITAL®, TAKATA Pharmaceutical Co., Ltd., Saitama) (*n* = 2) during the study period and those with confirmed medication noncompliance based on the electronic medical records (n = 2) were excluded. Patients were defined as full analysis set (FAS) and a flow chart of patient selection was drafted (Fig. 1). Patient demographic data included sex, age, body weight, PB dose, number of daily PB doses, PB dose per body weight, serum PB concentration, dosage formulation, administration route, serum albumin (ALB), aspartate aminotransferase (AST), alanine aminotransferase (ALT), lactase dehydrogenase (LDH), γ-glutamyl transpeptidase (γGTP), blood urea nitrogen (BUN), serum creatinine (Scr), and causative disorder. The medication history of drugs that potentially interact with PB, which might affect serum PB concentration (i.e., amiodarone, fluconazole, miconazole, disulfiram, fluvastatin, fluvoxamine, metronidazole, voriconazole, valproic acid (VPA), and stiripentol) [7, 8], was also assessed.

This study was conducted in accordance with the Declaration of Helsinki and its amendments, and was approved by the Clinical Research Ethics Review Committee of Mie University Hospital (No. H2020–026).

### Serum PB samples and assay methods

In each study, serum PB samples were separated from whole blood by centrifugation at 1700×g for 10 min at 19–26 °C using serum separation tube, and the total PB concentration was measured immediately. Total serum PB concentrations were determined using a chemiluminescent immunoassay reagent and a TDx® FLx (Abbott Diagnostics, Chicago, IL, U.S.A.) from 2007 to 2013 and ARCHITECT® i1000SR (Abbott Laboratories, Chicago, IL) from 2014 to 2019. The quantitative range of PB for both instruments was 1.1–80 μg/mL.

### Patient subgroups

As indicated in Fig. 1, the following 6 subgroups were established to evaluate the causes of the serum PB concentration changes, including the concomitant use of a drug that potentially interacts with PB (subgroups 1 and 2), dosage formulation, and administration route (subgroups 3–6). Subgroup 1 (*n* = 46) had patients without interacting drugs while subgroup 2 (*n* = 14) had patients with interacting drugs. Patients in subgroups 3, 4, 5, and 6 did not take the interacting drugs. Other characteristics of each subgroup were: subgroups 3 (*n* = 8) and 4 (*n* = 9) had patients receiving PB elixir via oral route and enteral tube, respectively, and subgroups 5 (n = 9) and 6 (*n* = 20) had patients receiving PB powder via oral route and enteral tube, respectively.

### Statistical analyses

Statistical analyses were performed using IBM SPSS Statistics version 25.0 (IBM, Ltd., Tokyo). Categorical and continuous variables on patient background were summarized as median [first quartile-third quartile] and frequency (percentage), respectively. Simple linear regression analysis was conducted to investigate the variation factors of the weight-corrected PB C/D ratio, and standardized partial regression coefficient was calculated.

Multiple linear regression analysis using the forward stepwise selection method and the optimal multiple regression equation was conducted for the significant factors related to the weight-corrected PB C/D ratio observed in the simple linear regression analysis. In this analysis, sex was coded as 1 = male 2 = female, formulation was 1 = PB elixir 2 = PB powder, interacting drug use was 1 = no 2 = yes, and administration route was 1 = oral route 2 = enteral tube, respectively.

For the subgroup analyses, the data are presented as mean ± standard deviation and were analyzed using Student’ s t-test or one-way analysis of variance (ANOVA). A *p*-value of < 0.05 was considered to indicate statistical significance.

## Results

### Patients

Based on the inclusion and exclusion criteria, 60 patients were enrolled in the retrospective study as FAS (Fig. 1). During the study period, the elixir and powder of PB were administered to 21 and 39 patients, respectively. All serum PB concentrations of the study subjects were within the calibration curve range. The patient characteristics are summarized in Table 1. The median [first quartile-third quartile] age and body weight of the study patients were 1.0 (0.6–2.5) years and 8.2 (4.7–11.7) kg, respectively. The median daily PB dose, number of daily PB doses, and amount of PB dose per body weight were 40.0 (20.0–60.0) mg/day, 2.0 (2.0–2.0) times/day, and 2.6 (1.9–3.7) mg/kg/time, respectively. The median serum concentration of PB was 16.0 (11.2–25.0) μg/mL. For the concomitant use of drugs that potentially interact with PB, the number of patients receiving VPA and amiodarone was 13 (22%) and 1 (2%), respectively. No other drugs were not identified as the interacting drugs of PB including typical CYP inducers in this study.

**Table 1:** Baseline characteristics of the patients

	FAS (*n* = 60)	PB elixir (*n* = 21)		PB powder (*n* = 39)	
		Oral route (*n* = 8)	Enteral tube (*n* = 13)	Oral route (*n* = 14)	Enteral tube (*n* = 25)
Male (%)	33 (55)	4 (50)	8 (62)	6 (43)	15 (60)
Age (years)	1.0 [0.6–2.5]	1.0 [0.7–1.9]	0.8 [0.2–1.0]	0.9 [0.5–2.5]	2.0 [1.0–3.2]
Body weight (kg)	8.2 [4.7–11.7]	8.1 [5.9–11.6]	4.0 [2.8–8.1]	8.8 [6.2–11.5]	8.8 [4.8–14.3]
PB dose (mg/day)	40.0 [20.0–60.0]	40.0 [31.0–60.0]	16.0 [9.8–23.2]	50.0 [27.5–80.0]	50.0 [19.0–80.0]
Number of daily PB doses (times/day)	2.0 [2.0–2.0]	2.0 [2.0–2.0]	2.0 [1.1–2.5]	2.0 [1.5–2.0]	2.0 [2.0–2.0]
PB dose per body weight (mg/kg/time)	2.6 [1.9–3.7]	2.6 [2.1–4.3]	2.0 [1.1–2.5]	2.7 [1.9–3.6]	2.9 [2.2–4.7]
Serum PB concentration (μg/mL)	16.0 [11.2–25.0]	15.4 [11.1–20.1]	15.7 [12.7–18.8]	11.4 [10.1–23.7]	17.4 [9.9–34.9]
ALB (g/dL)	4.1 [3.7–4.4]	4.2 [3.3–4.6]	4.0 [3.5–4.4]	4.3 [3.8–4.5]	4.0 [3.7–4.4]
AST (U/L)	35.9 [28.4–53.9]	34.9 [21.6–46.5]	46.0 [31.5–63.5]	35.0 [28.7–50.5]	39.0 [30.0–75.9]
ALT (U/L)	20.8 [15.0–39.8]	19.2 [12.5–25.8]	29.8 [13.2–35.5]	22.6 [16.5–42.6]	29.6 [15.7–52.5]
LDH (U/L)	272.6 [222.4–334.2]	264.5 [212.5–312.6]	255.0 [243.0–271.5]	320.7 [253.4–388.0]	277.0 [225.5–367.7]
γGTP (U/L)	69.9 [28.8–170.9]	30.5 [23.5–93.0]	65.0 [31.5–289.5]	39.5 [27.7–57.5]	76.3 [34.0–172.5]
BUN (mg/dL)	8.9 [5.8–11.9]	8.2 [5.2–13.0]	9.0 [3.5–9.6]	6.1 [5.4–8.5]	9.4 [6.3–13.5]
Scr (mg/dL)	0.2 [0.2–0.3]	0.2 [0.2–0.3]	0.2 [0.1–0.3]	0.2 [0.2–0.3]	0.2 [0.1–0.3]
**Interacting drug use**					
VPA (%)	13 (22)	0 (0)	3 (23)	5 (36)	5 (20)
Amiodarone (%)	1 (2)	0 (0)	1 (8)	0 (0)	0 (0)
Causative disorder *including duplication					
Fever convulsion (%)	11 (18)	2 (25)	3 (23)	2 (14)	4 (16)
Epilepsy (%)	18 (30)	2 (25)	3 (23)	4 (29)	9 (36)
Acute encephalitis/acute encephalopathy (%)	4 (7)	1 (13)	2 (15)	0 (0)	1 (4)
Intracranial bleeding (%)	9 (15)	1 (13)	1 (8)	5 (36)	2 (8)
Hypoxic-ischemic encephalopathy (%)	16 (27)	1 (13)	7 (54)	3 (21)	5 (20)
Hyperammonemia (%)	2 (3)	1 (13)	0 (0)	0 (0)	1 (4)
Brain tumor (%)	4 (7)	1 (13)	0 (0)	2 (14)	1 (4)
Angiocholitis (%)	2 (3)	1 (13)	0 (0)	0 (0)	1 (4)
Not clear (%)	2 (3)	0 (0)	0 (0)	0 (0)	2 (8)

Data are shown as frequency (percentage) or median [first quartile-third quartile]

PB phenobarbital, FAS full analysis set, ALB serum albumin, AST aspartate aminotransferase, ALT alanine aminotransferase, LDH lactate dehydrogenase, γGTP γ-glutamyl transpeptidase, BUN blood urea nitrogen, Scr serum creatinine, VPA valproic acid

### Simple linear regression analysis

**Fig. 2:**
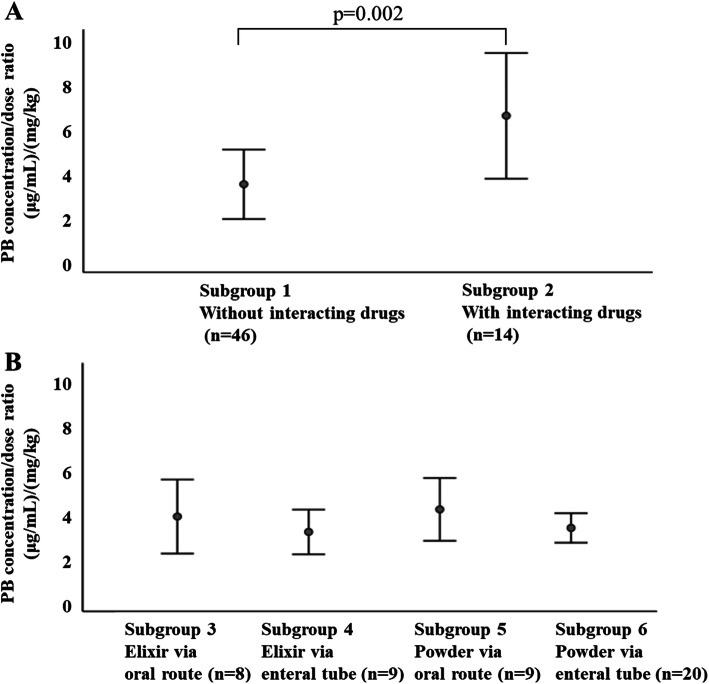
Difference in the weight-corrected PB C/D ratio between groups. (A) Effect of the concomitant use of the interacting drugs (VPA or amiodarone), (B) Effect of administration route and dosage formulation

Simple linear regression analysis was performed using the weight-corrected PB C/D ratio as the dependent variable and patient background as the explanatory variable. Table 2 shows the results of standardized partial regression coefficient (β), 95% confidence interval (CI), and *p*-value of a test on standardized partial regression coefficient. The significant variables were age (β = 0.390, *p* = 0.002), interacting drug use (β = 0.516, *p* < 0.001), body weight (β = 0.443, p < 0.001), and LDH (β = 0.259, *p* = 0.045). As shown in Fig. 2a, a significant difference in the PB C/D ratio was observed between subgroups 1 and 2, which were classified by interacting drug use (*p* = 0.002). There were no significant correlations between the PB C/D ratio and the other factors investigated.

**Table 2:** Simple linear regression analysis for the weight-corrected PB C/D ratio

	β	95% CI	p-value
Male	0.094	−0.168 - 0.355	0.477
Age	0.390	0.147–0.632	0.002
Body weight	0.443	0.207–0.679	< 0.001
PB dose	0.163	−0.097 - 0.422	0.214
Number of daily PB doses	0.116	−0.145 - 0.377	0.376
ALB	0.133	−0.128 - 0.393	0.312
AST	0.151	−0.109 - 0.411	0.249
ALT	−0.135	−0.395 - 0.125	0.304
LDH	0.259	0.006–0.513	0.045
γGTP	0.249	−0.005 - 0.504	0.055
BUN	−0.037	−0.300 - 0.226	0.779
Scr	0.037	−0.226 - 0.300	0.780
Interacting drug use (VPA or amiodarone)	0.516	0.291–0.741	< 0.001
Dosage formulation	−0.225	−0.481 - 0.032	0.085
Administration route	−0.134	−0.395 - 0.126	0.306

C/D serum concentration/dose, VPA valproic acid, PB phenobarbital, ALB serum albumin, AST aspartate aminotransferase, ALT alanine aminotransferase, LDH lactate dehydrogenase, γGTP γ-glutamyl transpeptidase, BUN blood urea nitrogen, Scr serum creatinine β standardized partial regression coefficient, CI confidence interval

Simple linear regression analysis was performed using the weight-corrected PB C/D ratio as the dependent variable and patient background as the explanatory variable

### Multiple linear regression analysis

Table 3 shows the results of the regression equation by multiple linear regression analysis (forward stepwise selection method). The explanatory variables were selected according to a significant correlation with the weight-corrected PB C/D ratio by single linear regression analysis. This analysis revealed that the factors for the PB C/D ratio in pediatric patients were interacting drug use (standardized partial regression coefficient [β], 0.543, *p* < 0.001), body weight (β = 0.346, p < 0.001), and LDH (β = 0.272, *p* = 0.006). The following significant regression equation was found to predict the PB C/D ratio, (an adjusted coefficient of determination = 0.530, p < 0.001):

**Table 3:** Multiple linear regression analysis for the weight-corrected PB C/D ratio

	α	β	95% CI	p-value	VIF
Constant	-2.026				
Body weight	0.185	0.346	0.139 - 0.533	<0.001	1.039
LDH	0.004	0.272	0.086 - 0.478	0.006	1.037
Interacting drug use (VPA or amiodarone)	2.961	0.543	0.306 - 0.700	<0.001	1.042

C/D serum concentration/dose, VPA valproic acid, PB phenobarbital, LDH lactate dehydrogenase, α partial regression coefficient, β standardized partial regression coefficient, CI confidence interval, VIF variance inflation factor

Multiple linear regression analysis (forward stepwise selection method) and regression equation was found.

The explanatory variables were selected according to a significant correlation with the weight-corrected PB C/D ratio using single linear regression analysis.

Weight-corrected PB C/D ratio = -2.026 + 2.961 × [interacting drug use: (1=no, 2=yes)] + 0.185 × [body weight (kg)] +0.004 × [LDH (U/L)]

Weight-corrected PB C/D ratio = − 2.026 + 2.961 × [interacting drug use: (1 = no, 2 = yes)] + 0.185 × [body weight (kg)] + 0.004 × [LDH (U/L)].

The concomitant use of the potential interacting drugs against PB had the largest effect on the prediction equation of the weight-corrected PB C/D ratio. In contrast, the subgroup analysis classified by the administration route and dosage formulation (subgroups 3–6) showed no significant differences based on the weight-corrected PB C/D ratio (Fig. 2b).

## Discussion

A simple linearity is reported to be observed between serum PB concentration and dose in the concentration range of 10–40 μg/mL [9]. Further, the bioavailability of the elixir and powder of PB is almost 100% [10]. However, this finding was observed with adult data. Over the neonatal and infant periods, the bioavailability of PB is estimated to be less than 50%, and the systemic clearance of PB follows a sigmoidal pattern [11]. Age-related drug disposition changes derived from liver and kidney function, plasma protein binding rate, tissue distribution volume, and metabolic enzyme activity contribute to the pharmacokinetic differences in the pediatric population [12–15]. Gastric pH is also increased in the pediatric population and reaches adult pH values by 2 years of age. PB is a weak acid product and its absorption rate in pediatric population is lower than that in older children and adult [16]. Since intestinal motility depends on feeding habits, drug administration that elevate gastric pH, and intestinal bacterial growth [16, 17], concomitant use of proton-pump inhibitors (PPI) or histamine type 2 (H_2_) receptor antagonists may cause the decreased intestinal absorption of PB in pediatric patients. In this study, there were only 2 patients who concomitantly received PPI or H_2_ receptor antagonists during the study period. Indeed, the weight-corrected PB C/D ratios [3.48 and 3.05 (μg/ml)/(mg/kg)] in the two patients were relatively lower than the mean ± standard deviation value [4.05 ± 0.30 (μg/ml)/(mg/kg)] in the patients without PPI or H_2_ receptor antagonists. The bioavailability of phenytoin and ganciclovir, both of which are weak acid drugs, was also reported to be lower in the pediatric population than in adults [18, 19]. Additional analysis is needed to reveal the contribution of gastric pH to the intestinal absorption of PB.

Several causes of serum PB concentration fluctuations, including dosage formulation, have been reported. To our knowledge, our study is the first to comprehensively analyze the variation factors of serum PB concentration with drug-drug interactions, dosage formulation, and administration route. We demonstrated that the concomitant use of the drugs that potentially interact with PB significantly affected the PB C/D ratio in pediatric patients. PB is partly metabolized in the liver at 45–65% mainly by CYP 2C9, which matures significantly within 1 year of age [8, 20]. In addition, the main metabolite is further conjugated with gluconic acid [20]. For instance, the unbound oral clearance of (S)-warfarin, a typical substrate of CYP2C9, in the pediatric population was reported to be significantly lower than that in adults [21]. Immature CYP2C9 activity in neonates may affect the pharmacokinetic differences in its substrates between neonates and adult patients. On the other hand, clearance of PB has been reported to be higher in children than in adults [22]. Similarly, in our study, simple linear regression analysis showed a positive correlation between PB C/D ratio and age. These evidences provide the insight that drug interaction between PB and its inhibitors should be predicted under the consideration of patient’s age.

VPA inhibits N-glucoside excretion, which contributes 16.2% of PB metabolism [23]. Further, the serum PB concentration in patients with VPA was reported to be 32% higher than that in patients without VPA [23]. In our study, one of the 14 patients who received drugs that might interact with PB was also administered amiodarone, and his weight-corrected C/D ratio of 4.2 was relatively higher than that in the patients without interacting drugs (mean weight-corrected C/D ratio: 3.79). For amiodarone, desethylamiodarone (DEA), a principal metabolite of amiodarone, inhibits CYP1A1, CYP1A2, CYP2A6, CYP2B6, CYP2C9, CYP2C19, CYP2D6, and CYP3A4 [24]. DEA has been reported to potently inhibit CYP2C9-mediated warfarin hydroxylation both in vitro and in vivo [25]. Although there have been no reports demonstrating the elevation of serum PB concentration due to the concomitant use of amiodarone, the AUC of (S)-warfarin administered concomitantly with amiodarone increased to 211% compared to that of (S)-warfarin alone [25].

In the present study, multiple linear regression analysis showed that body weight and LDH were risk factors that affected the PB C/D ratio (Table 3). In neonates, extracellular fluid accounts for 40–50% of body weight, which is quite higher than that in adults (30%) [26]. In addition, the reported protein binding rate of PB in newborns less than 2 weeks (37%) is lower than that in adults (51%) [27]. Such changes are partly related to the differences of the volume of distribution of PB between pediatric and adult patients (pediatric patient: 0.9 L/kg [28] vs. adult: 0.7 L/kg [29]). Therefore, a decrease in the proportion of water composition and PB tissue distribution with growth during childhood might be associated with an increase in the weight-corrected C/D ratio of PB. However, more evidence will be needed to clarify the contribution of the water composition to the C/D ratio of PB. LDH is an enzyme present in almost all body tissues. Elevated LDH is often observed in liver disease, anemia, heart attack, bone fractures, muscle trauma, cancers, and infections [30]. Since AST and ALT were not extracted as risk factors by simple and multiple linear regression analyses in our study, liver dysfunction might not affect the increase in the PB C/D ratio. Although the influence of LDH on the PB C/D ratio was less than the existence of drug-drug interactions, further research is needed to confirm the relationship between LDH level and serum PB concentration.

In this study, we found no relationship between the PB C/D ratio and dosage formulation (elixir vs. powder) in contrast to the previous report [5]. The previous report showed that there were significant differences in the weight-corrected PB C/D ratio between the elixir and powder groups [5.7 ± 2.6 vs. 3.6 ± 2.1 (μg/ml)/(mg/kg) (*p* = 0.044)]. Our study is quite similar to the previous study in point of the number of patients, age, and ethics. However, analysis and evaluation methods differ from each other. In addition, the difference of PB formulation between powder and elixir was a focal point in the previous report and the concomitant use of drugs, the difference of administration route, and liver and kidney functions were not included as confounding factors. Since our study performed a multivariate analysis with pediatric care issues, including drug-drug interactions, more reliable data were considered to be provided.

Because dosage formulation with easy dose adjustment is desirable for the pediatric population, liquid and powder formulations are commonly selected as suitable formulations for use in pediatric medical care. As shown in Fig. 2b, the dosage formulation and administration route did not largely affect the serum PB concentration when VPA or amiodarone was not administered concomitantly. In the present study, 4 patients switched from the elixir to the powder PB formulation and had serum PB concentration before and after switching. In a patient concomitantly using VPA, the PB C/D ratio decreased by 2.33 after the PB formulation was switched (C/D ratio: elixir 9.09, powder 6.76); this was the largest change among the 4 patients. Meanwhile, the absolute values of the differences in the PB C/D ratio in the other 3 patients without VPA or amiodarone were 1.30, 0.95, 0.57, respectively. This result demonstrates the importance of considering drug-drug interactions against PB in pediatric drug therapy.

This study had some limitations. First, we could not evaluate the data by age because of the limited number of patients. As a result, a large variability in the absolute value of the weight-corrected C/D ratio (Fig. 2a, b) was observed. Therefore, a more age-specific survey would be required. Additionally, only 14 patients used drugs that might affect their serum PB concentration.

This study is a retrospective chart review, and prospective studies are needed to determine the importance of drug-drug interactions in PB administration to pediatric patients.

## Conclusion

Herein, the concomitant use of VPA or amiodarone was found to largely affect the PB C/D ratio in pediatric patients. However, switching from the elixir to the powder PB formulation and administration route did not have a significant effect on the PB C/D ratio. Our study can be viewed as a valuable attempt in the evaluation of clinical data of pediatric patients. Further, our results could provide important perspectives in pediatric drug therapy where elixir and powder formulations are administered via the oral route and an enteral tube.

## Data Availability

All data generated or analyzed during this study are included in this published article.

## Abbreviations

PB: phenobarbital
C/D: serum concentration/dose
FAS: full analysis set
ALB: serum albumin
AST: aspartate aminotransferase
ALT: alanine aminotransferase
LDH: lactase dehydrogenase
γGTP: γ-glutamyl transpeptidase
Scr: serum creatinine
BUN: blood urea nitrogen
ANOVA: analysis of variance
VPA: valproic acid
β: standardized partial regression coefficient
CI: confidence interval
